# Speech Production From a Developmental Perspective

**DOI:** 10.1044/2019_JSLHR-S-CSMC7-18-0130

**Published:** 2019-08-29

**Authors:** Melissa A. Redford

**Affiliations:** a Linguistics Department, University of Oregon, Eugene

## Abstract

**Purpose:**

Current approaches to speech production aim to explain adult behavior and so make assumptions that, when taken to their logical conclusion, fail to adequately account for development. This failure is problematic if adult behavior can be understood to emerge from the developmental process. This problem motivates the proposal of a developmentally sensitive theory of speech production. The working hypothesis, which structures the theory, is that feedforward representations and processes mature earlier than central feedback control processes in speech production.

**Method:**

Theoretical assumptions that underpin the 2 major approaches to adult speech production are reviewed. Strengths and weaknesses are evaluated with respect to developmental patterns. A developmental approach is then pursued. The strengths of existing theories are borrowed, and the ideas are resynthesized under the working hypothesis. The speech production process is then reimagined in developmental stages, with each stage building on the previous one.

**Conclusion:**

The resulting theory proposes that speech production relies on conceptually linked representations that are information-reduced holistic perceptual and motoric forms, constituting the phonological aspect of a system that is acquired with the lexicon. These forms are referred to as exemplars and schemas, respectively. When a particular exemplar and schema are activated with the selection of a particular lexical concept, their forms are used to define unique trajectories through an endogenous perceptual–motor space that guides implementation. This space is not linguistic, reflecting its origin in the prespeech period. Central feedback control over production emerges with failures in communication and the development of a self-concept.

Speech motor control allows for flexible, fast, and precise coordination of speech articulators to achieve a motor goal. Adult performance in auditory feedback perturbation experiments suggests not only sensitivity to deviations between, say, an intended vowel and the acoustics of the vowel produced but also an ability to compensate for these deviations with fine motor adjustments that can raise or lower a particular formant frequency by as little as 50 Hz (see, e.g., Katseff, Houde, & Johnson, 2012; MacDonald, Goldberg, & Munhall, 2010). It is perhaps not surprising that this kind of fine-grained spatiotemporal control over articulation develops slowly. Large gains in speech motor skill are made during the first few years of life, but adultlike control is not achieved until mid-adolescence. Evidence for this claim dates back to Kent and Forner (1980), who pointed out that temporal variability in young school-aged children's segmental durations is higher than in adults' speech and that this remains true until 12 years of age (see also Lee, Potamianos, & Narayanan, 1999; B. L. Smith, 1992). These acoustic findings were later supplemented with kinematic ones, which validated the interpretation of greater temporal variability in children's speech as the result of immature articulatory timing control (Green, Moore, Higashikawa, & Steeve, 2000; Sharkey & Folkins, 1985; A. Smith & Goffman, 1998). A. Smith and Zelaznik (2004) followed up on this work with older children and showed that articulatory timing control is not fully mature until mid-adolescence. So, given the protracted development of speech motor control, why can we more or less understand what children are saying when they first begin to use words at about 12 months of age? Also, even more strikingly, how is it possible that 3-year-old children seem to never stop talking when their speech motor skills are still so immature? The answer put forward in this review article is that feedforward processes mature earlier than central feedback control processes.

More specifically, the argument developed herein is that speech production relies on conceptually linked representations that are abstract (i.e., information-reduced) holistic perceptual and motoric forms. These forms constitute the phonological aspect of the lexicon. The perceptual phonological forms are exogenous representations. They are exemplars that are acquired with lexical concepts beginning around 9 months of age. The motoric phonological forms are endogenous representations. They are schemas that begin to be abstracted around 12 months of age with first word productions. When a particular exemplar and schema are activated with the selection of a particular concept, their forms are used to define unique trajectories through an endogenous perceptual–motor space that guides implementation. This space is not linguistic; its processes are entirely free from conceptual information. The absence of conceptual information reflects the origin of this space in the prespeech period when infants' vocal explorations create the first linkages between perceptual and motoric trajectories.

By hypothesis, schemas are modified through developmental time as central feedback control is incorporated into the production process. This is because the act of speaking indirectly modifies schemas via the same process used to first abstract them. The onset of high-level predictive feedback control emerges with communication failures. These failures are assumed to significantly increase with vocabulary size due to homophony, motivating a shift in the production system toward exemplar representations around 18 months of age. The shift drives the emergence of an internal loop that matches the (projected) perceptual consequences of self-productions against targeted exemplar representations. Selective attention to auditory feedback develops later during the preschool years with the emergence of self-concept. At this point, the child begins to focus on sound production per se in addition to communication. The latter hypothesis could explain why literacy acquisition becomes possible around the age of 5 years and why direct intervention for speech sound disorders also becomes effective at this age.

The argument outlined above is in fact a general theory of speech production that is developmentally sensitive. The theory combines those aspects of existing adult-focused theories that best accommodate acquisition to define whole-word production at different stages of development from infancy to childhood on into adulthood. This developmentally sensitive theory of speech production is further motivated below. This motivation begins with a review of adult-focused theories. A major point of the review will be that the two major approaches to speech, the ecological dynamics and information-processing approaches, lead to different emphases regarding the type of feedforward information used in production (motoric vs. perceptual) and to different views on the type of feedback control processes engaged during execution (peripheral vs. central). I will argue that the holistic motoric representations that drive production in the ecological dynamics approach are consistent with functional approaches to child phonology and better account for young children's speech patterns than the discrete perceptual representations that drive production in the information-processing approach. Nonetheless, the information-processing assumption of distinct production and perception systems is embraced in the developmentally sensitive theory of speech production that I put forward because central feedback control is deemed necessary to account for the evolution of children's speech patterns from first words to adultlike forms.

## Adult-Focused Theories of Speech Production

**Figure 1.**
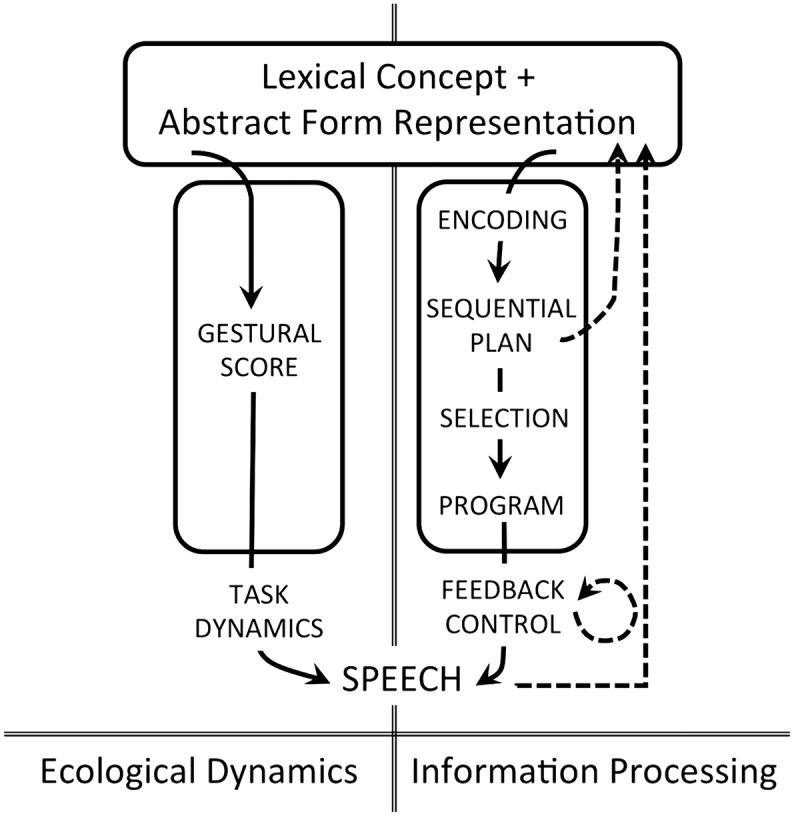
The ecological dynamics and information-processing approaches to speech production both assume three major levels of analysis: a phonological level where abstract form representations are associated with conceptual meaning, a speech plan level where abstract forms are elaborated for implementation, and an implementation level where articulatory action is formulated and adjusted in real time to achieve the plan. The two approaches otherwise adopt very different fundamental assumptions, resulting in different theories of representation, sequencing, and control. In particular, the ecological dynamics approach emphasizes speech as action and assumes gestalt articulatory representations, emergent sequential structure, and self-organized articulation. In contrast, the information-processing approach emphasizes the importance of discrete elements and assumes executive control over sequencing and implementation, thus promoting a strong role for perception in production while assuming that the two processes are distinct. Solid lines with arrows represent feedforward processes; dotted lines with arrows represent feedback processes.

Adult-focused theories of speech production assume the activation of an abstract phonological plan that is then rendered in sufficient phonetic detail for the sensorimotor system to activate speech movements (e.g., Browman & Goldstein, 1992; Dell, 1986; Garrett, 1988; Goldrick, 2006; Goldstein, Byrd, & Saltzman, 2006; Guenther, 1995; Keating & Shattuck-Hufnagel, 2002; Roelofs, 1999; Turk & Shattuck-Hufnagel, 2014). The detailed phonetic plan is known as a *speech plan*. It contains or directly activates linguistic representations that provide relevant feedforward information for implementation. The representations and type of feedback control processes used in production differ according to the theoretical approach taken. Here, the two main approaches to speech production are reviewed: the ecological dynamics approach and the information-processing approach (see Figure 1). These approaches represent an amalgam of different theories, hence the generic labels. The different sets of theories emerge from two fundamentally different approaches to human cognition—an ecological-embodied approach versus a representation-based information-processing approach, which are briefly described next.

Richardson, Shockley, Fajen, Riley, and Turvey (2009) outline the tenets of an ecological-embodied approach in contrast to the assumptions of an information-processing approach as follows. In an ecological-embodied approach, behavior is emergent and self-organized, which is to say behavior is not planned or controlled (pp. 170–173). Perception and action are viewed as continuous and cyclic and thus functionally united (pp. 173–175). In particular, the concept of affordances assumes that the objects of perception provide information about action possibilities (pp. 178–182). The theory of direct perception assumes that these useful objects are wholly conveyed by sensory input (pp. 176–178). This means that knowledge is simply extracted from the environment within which the individual lives and moves (pp. 167–170).

The ecological-embodied view of knowledge contrasts with the information-processing view where knowledge emerges from learned associations, which give rise to mediating representations. These representations are knowledge in the information-processing approach. This view of knowledge follows from other assumptions: Individuals are separate from their environment, the mind is separate from the body, and action is separate from perception. Overall, representational and computational processes are “lifted away from the organism–environment system and…studied on their own, permitting cognitive scientists to proceed whereas other specialists work to understand the body and environment of the knower” (Richardson et al., 2009, pp. 161–162). This approach to human cognition is likely more familiar to readers than the ecological-embodied approach because it has provided the philosophical foundation for much of mainstream cognitive sciences in North America, including linguistics and psychology, since the “cognitive revolution” in the 1950s (see Mandler, 2007, Chap. 10). The assumptions of this approach are detailed in Newell and Simon's (1972) classic book, *Human Problem Solving*.

The information-processing approach has resulted in the modular study of language (e.g., syntax vs. phonology) and in a sharp division of expertise between those who study language and those who are interested in speech production (e.g., phonology vs. phonetics). Among the latter, those who adhere closely to the approach often focus on the translation problem that follows from their computational view, for example, the problem of how discrete phonological elements are transformed into continuous speech action (see, inter alia, Bladon & Al-Bamerni, 1976; Keating, 1990; MacNeilage, 1970; Recasens, 1989; Stevens & Blumstein, 1981; Wickelgran, 1969). This focus also structures psycholinguistic models of production that posit multiple processing stages to generate production units (e.g., Dell, 1986; Garrett, 1988; Goldrick, 2006; Levelt, 1989; Roelofs, 1999), a generic version of which is presented in the right-hand panel of Figure 1. Models of speech motor control that have discrete elements as goals emphasize feedback control to ensure accurate implementation of these elements in speech movement (e.g., Abbs & Gracco, 1984; Hickok, 2012; Houde & Nagarajan, 2011; Lindblom, Lubker, & Gay, 1979; Niziolek, Nagarajan, & Houde, 2013; Perkell, Matthies, Svirsky, & Jordan, 1993; Tourville & Guenther, 2011).

In contrast to the information-processing approach, the ecological-embodied approach has been mainly applied to the study of speech (Best, 1995; Browman & Goldstein, 1992; Fowler, 1986; Galantucci, Fowler, & Turvey, 2006; Goldstein & Fowler, 2003; Kelso, Saltzman, & Tuller, 1986; Saltzman & Kelso, 1987; Saltzman & Munhall, 1989). The assumption of separate language and speech systems is thus preserved by default, and only speech processes are fully consistent with the tenets of an ecological-embodied approach. This entails no translation between higher level speech sound representations and lower level speech movement. Phonological forms are objects of both action and perception. These forms become increasingly elaborated when activated through self-organization rather than through planning. Thus, the flow from high to low is better conceived of as the emergence of speech form, which is mediated only by a linearized version of a nonlinear representation (i.e., a gestural score; see Figure 1, left). The specific assumptions of each approach to speech production are elaborated further below, beginning with the action-focused ecological dynamics approach.

### The Ecological Dynamics Approach

The ecological dynamics approach to speech production is best represented by articulatory phonology (Browman & Goldstein, 1992, and subsequent), a task-dynamic approach to articulation (Kelso et al., 1986; Saltzman & Kelso, 1987; Saltzman & Munhall, 1989), and by ecological theories of speech perception (Best, 1995; Fowler, 1986; Galantucci et al., 2006; Goldstein & Fowler, 2003) and speech sound acquisition (Best, 1995; Best, Goldstein, Nam, & Tyler, 2016). The fundamental unit of analysis is a vocal tract constriction that serves as an articulatory attractor. This unit is known as a *gesture*. Gestures are linguistic primitives, similar to distinctive features in generative theory, that emerge during development under the assumption that infants acquire “a relation between actions of distinct (articulatory) organs and lexical units very early in the process of developing language” (Goldstein & Fowler, 2003, p. 35; see also Best et al., 2016). Gestures are defined as “events that unfold during speech production and whose consequences can be observed in the movements of the speech articulators” (Browman & Goldstein, 1992, p. 156). More specifically, they are abstract representations of “the formation and release of constrictions in the vocal tract (*ibid*),” which are realized dynamically, thus giving them an event-like status. This status in turn confers intrinsic timing; that is, once activated, gestures take time to achieve a target vocal tract constriction and then time to move away from the constriction.

The assumption of intrinsic timing has a number of interesting theoretical consequences, several of which are compatible with a developmental perspective on speech production. Perhaps, the most important of these consequences is in the representation of sequential articulation (see, e.g., Browman & Goldstein, 1992; Fowler, 1980; Fowler & Saltzman, 1993; Kelso et al., 1986; Saltzman & Munhall, 1989). Gestures, like their distinctive feature counterparts in generative phonology, are always realized as part of a larger whole (i.e., a “molecule”). However, unlike distinctive features, the wholes are not bundled up into individual phonemes that must be sequenced during the production process. Instead, gestures participate in an articulatory gestalt that is, minimally, syllable sized. Moreover, all relevant gestures associated with a lexical entry are coactivated when that entry is selected for production (Browman & Goldstein, 1989, 1992; Goldstein et al., 2006). Put another way, the articulatory phonology view of lexical form representations is that these are holistic and motorically based. The developmentally sensitive theory I propose shares this view of lexical representation; I also argue for holistic, perceptually based form representations.

Under the ecological-embodied assumption of cyclic action, appropriate sequencing within a word is emergent. To understand emergent sequencing, consider, for example, the coordination of a single consonantal and vocalic gesture. Consonantal gestures are intrinsically shorter than vocalic gestures. They are also phased relative to one another: If the cyclic gestures are coordinated without a phase difference, a consonant–vowel syllable emerges; if they are 180° out of phase, a vowel–consonant syllable emerges (Browman & Goldstein, 1988; Goldstein et al., 2006; Nam, Goldstein, & Saltzman, 2009). These in-phase and antiphase relations are stable coordination patterns in motor systems (Haken, Kelso, & Bunz, 1985; Turvey, 1990). Of course, languages allow for consonant or vowel sequences that complicate stable coordination dynamics (e.g., consider the English word “sixths” among many, many others). Thus, gestural timing associated with individual words may be learned during speech acquisition and incorporated into a coupling graph, which is the lexical form representation in articulatory phonology (Goldstein & Fowler, 2003; Goldstein et al., 2006; Nam et al., 2009).

Note that the ecological dynamics conception of coordination also has implications for a theory of coarticulation, which is understood within this approach to speech production as coproduction (see Fowler, 1980). In contrast to information-processing approaches to coarticulation, dynamic formant trajectories and distributed spectral effects of rounding and nasalization and so on emerge directly from the representation; they are never due to a central executive that “looks ahead” to the next sound(s) while preparing the current one. This view of coarticulation appears to be more compatible with developmental findings on coarticulation than the information-processing view, a point to which I return later.

When words are selected for production, their coupling graphs give rise to linearized gestural scores (see, inter alia, Goldstein et al., 2006). These scores meet the generic definitions of both a speech plan and a motor program. They are plans in that they specify, abstractly, the relative timing and duration of specific speech actions. They are programs in that they drive these actions directly via task dynamics (Saltzman & Munhall, 1989). The dynamic transformation from coupling graph to gestural score means that there is no speech planning in the ecological dynamic approach to speech production; there are only speech plans that serve also as phonological representations. I make a similar assumption in the developmentally sensitive theory proposed herein.

During the implementation stage of the production process, gestures represent motor goals (Fowler & Saltzman, 1993; Löfqvist, 1990). Articulators self-organize to effect these goals. Self-organization is based in large part on functional synergies that stabilize over developmental time to become part of the motor control system (see, e.g., A. Smith & Zelaznik, 2004). In other words, gestures give rise to a type of functional motor unit of coordination (i.e., a “coordinative structure”). Peripheral perceptual feedback provides relevant context information to subcortical structures and the peripheral nervous system for goal achievement (see, e.g., Saltzman & Munhall, 1989, p. 48) and to automatically compensate for perturbations (see, e.g., Abbs & Gracco, 1984). In this way, there is no real control over production in the sense of cortically mediated adjustments to movement direction and velocity. Whereas this view of implementation and its development can account for infant vocalizations and early speech attempts and for the overall slow development of speech motor skills, I argue below that the strong evidence from adult speech for cortically mediated control over production must be incorporated into a developmentally sensitive theory of speech production to account for phonological change through developmental time.

In summary, an ecological dynamics approach to speech production assumes an entirely feedforward process. Motor goals are articulatory and event-like and are phased relative to one another in articulatory gestalt representations that are linked to conceptual information in the lexicon. Sequential structure and coarticulatory overlap emerge from gestural dynamics. Production itself is a self-organized process. Thus, the approach eschews the concept of central control over speech production based on first principles.

### The Information-Processing Approach

The information-processing approach to speech production is best represented by mainstream psycholinguistic theories of language production (e.g., Dell, 1986; Garrett, 1988; Goldrick, 2006; Roelofs, 1999), phonetically informed theories of implementation (e.g., Guenther, 1995; Guenther & Perkell, 2004; Keating & Shattuck-Hufnagel, 2002; Turk & Shattuck-Hufnagel, 2014), and by prediction-based models of speech motor control (e.g., Hickok, 2012; Houde & Nagarajan, 2011; Niziolek et al., 2013; Tourville & Guenther, 2011). In this approach, phonological representations mediate between perception and production. They are abstract and symbolic.

The phoneme—a categorical and discrete element—is often the fundamental unit of analysis in this approach. The emphasis on phonemes is due to a modeling focus on speech errors (e.g., Bock & Levelt, 2002; Dell, 1986; Garrett, 1988; Levelt, 1989; Roelofs, 1999), which are best described with reference to segmental structure (see also MacKay, 1970; Shattuck-Hufnagel & Klatt, 1979). These modeling efforts have led to the psycholinguistic assumption that segment sequencing is an active process during production (see, inter alia, Bock & Levelt, 2002; Dell, 1986; Garrett, 1988; Levelt, 1989; Roelofs, 1999). This process has come to be known as *phonological encoding* (see Figure 1, right). Theories diverge on how encoding happens, but once encoded, all theories recognize that the phonemic string must be further specified before it can be used as a plan for output. In Levelt's (1989) highly influential model, the string is metrically chunked for output, allowing for specification of positional information via allophone selection; for example, the aspirated variant of the voiceless alveolar stop is chosen for *tab* (i.e., [tʰæb]), the unreleased variant is selected for *bat* (i.e., [bæt̚]), and the stop is replaced by a flap in *batter* (i.e., [bæɾɚ]). From a developmental perspective, the mainstream assumption of phonological and phonetic encoding complexifies speech acquisition since it predicts that infants must learn a symbolic system and the computational steps necessary to translate symbolic representations into action plans.

Once a phonological string has been phonetically encoded, it can be implemented. Implementation can mean the appropriate selection of a syllable-sized motor program from a mental syllabary (e.g., Bohland, Bullock, & Guenther, 2010; Guenther, Ghosh, & Tourville, 2006; Levelt, 1989) or careful specification of articulatory timing information (e.g., Keating, 1990; Turk & Shattuck-Hufnagel, 2014). Either way, discrete phones remain high-level motor goals during execution. These goals are conceived of specifically as speech sound categories (e.g., Guenther, 1995; Hickok & Poeppel, 2000; Johnson, Flemming, & Wright, 1993; Lindblom, 1990; Lindblom et al., 1972) or more generally as perceptual categories (e.g., Perkell, Matthies, Svirsky, & Jordan, 1995; Savariaux, Perrier, & Orliaguet, 1995; Schwartz, Boë, Vallée, & Abry, 1997). Importantly, the goals remain nonoverlapping even in high-frequency combinations when, through repeated practice, they may be stored together as part of a larger chunk (see, e.g., Bohland et al., 2010, p. 1505). This view stands very much in contrast to the ecological dynamics view where chunks are articulatory gestalts composed of overlapping gestures/articulatory events. The assumption of discrete goals also requires computationally intensive accounts of coarticulation, especially long-distance coarticulation, which is explained in the information-processing approach to result either from feature spreading at an early stage of encoding (e.g., Bladon & Al-Bamerni, 1976; Daniloff & Hammarberg, 1973; Recasens, 1989) or from planning for the articulation of individual phones within a well-defined window during a later stage of encoding (e.g., Guenther, 1995; Keating, 1990). These accounts wrongly predict the slow development of coarticulation (see below).

Although discrete perceptual speech motor goals are problematic from a development perspective, they are posited in the information-processing approach to explain “the exquisite control of vocal performance that speakers/singers retain for even the highest frequency syllables” (Bohland et al., 2010, p. 1509). Exquisite control of vocal performance requires the coordination of multiple independent speech articulators through time, each of which also has many degrees of movement freedom—another developmentally unfriendly computational problem. The coordination problem is solved in the information-processing approach by assuming central perceptual feedback control over articulatory movements—an assumption for which there is now abundant evidence.

Central feedback control means cortically mediated adjustments to articulation made with reference to perceptual goals in order to achieve on-target sound production. Of course, slow central processing of perceptual feedback presents a problem for perceptual feedback during real-time speech production (see, e.g., Lindblom et al., 1979; MacNeilage, 1970). Lindblom et al. (1979, p. 160) were the first to propose a viable solution to this problem. Specifically, they proposed that motor control does not rely on processing perceptual feedback per se but instead references the simulated perceptual results of planned action while execution unfolds. Lindblom et al. called this proposal *predictive encoding*, and with it, they foreshadowed the emphasis in current models of speech motor control where a copy of the output signal (= efference copy) is used to predict sensory outcomes (e.g., Hickok, 2012; Houde & Nagarajan, 2011; Niziolek et al., 2013; Tourville & Guenther, 2011) for error correction purposes (e.g., Tourville & Guenther, 2011) or real-time speech motor control (see, e.g., Niziolek et al., 2013). The proposal is supported by speakers' remarkable ability to correctly produce target sounds when normal articulation is disrupted.

Lindblom et al. (1979) proposed predictive encoding to account for their speakers' near-instantaneous adaptation to different bite-block manipulations during vowel production. Since then, many sophisticated perturbation experiments have been conducted (e.g., Katseff et al., 2012; Lametti, Nasir, & Ostry, 2012; MacDonald et al., 2010; Savariaux et al., 1995). These experiments provide strong evidence in favor of perceptual goals and for the role of central feedback control in speech production. Consider, for example, a study by Lametti et al. (2012), which investigated the effects of different types of perceptual feedback perturbations on the repetition of a target word, *head*. Somatosensory feedback was disrupted by a robot arm, which tugged randomly at the speakers' lower jaw, thereby disrupting the normal articulatory path for the target /ɛ/ vowel. Auditory feedback was perturbed by altering the speaker's own F1 upward in the direction of an /æ/ vowel. This real-time alteration was sent to the speaker via headphones. The results indicated that speakers counteracted the effects of perturbation through compensation to maintain the target, *head,* production. While the majority of speakers compensated more for auditory perturbations than somatosensory perturbations, some speakers showed the opposite effect and many adapted to both types of perturbations.

It has been argued that, whereas perturbation experiments provide evidence for error correction based on perceptual feedback, conclusions about real-time speech motor control are more dubious since the experimental findings require manipulations that create very unnatural speaking conditions (see, e.g., Guenther et al., 2006, p. 288). Yet, the basic behavior observed in perturbation experiments—speaker adjustments based on incoming perceptual information—is also observed in phonetic imitation experiments, which are significantly more natural. Instead of participants hearing their own perturbed speech, they simply repeat words that others have produced (e.g., Babel, 2012; Goldinger, 1998; Nielsen, 2011; Shockley, Sabadini, & Fowler, 2004). Just as in the perturbation paradigm, participants are found to make fine-tuned adjustments to their own speech in the direction of the input; for example, participants' production of voice onset time (VOT) in stop production is measurably changed when shadowing exposure to stop-initial words with substantially different VOT values than their own (Shockley et al., 2004). Moreover, behavior in these laboratory experiments also corresponds to the real-world language phenomenon of convergence (Giles & Powesland, 1997), where interlocutors begin to sound like one another over the course of an exchange. When speakers subconsciously “converge” on a set of phonetic features during an interaction, they are demonstrating that perceptual input informs online spoken language production (see, e.g., Babel, 2012). Thus, speakers' behavior in contrived and natural speaking conditions provides strong evidence for the importance of perceptual feedback during speech production. The developmentally sensitive theory proposed herein is meant to accommodate this evidence.

In summary, the information-processing approach emphasizes the importance of discrete elements and so assumes executive control over sequencing and implementation. This assumption entails a role for perception in production. The evidence for online vocal–motor adjustments based on self- and other- generated auditory information is especially strong and consistent with the hypothesis of central perceptual feedback control over speech production.

## Implications of Adult-Focused Theories for the Development of Speech Production

From a developmental perspective, the different approaches to speech production each has strengths and important limitations that were alluded to above. The main strength of the ecological dynamics approach is the central hypothesis that temporal relations between articulators are preserved as part of an articulatory gestalt lexical representation. This hypothesis, consistent with whole-word approaches to child phonology, provides a framework for understanding children's speech patterns. The strength of the information-processing approach is in recognizing the importance of perceptual feedback for tuning speech production. This emphasis is not only consistent with adult behavior; it also provides a powerful mechanism for learning and thus the ability to explain change over developmental time. These points are elaborated below with a focus on explaining children's speech patterns and developmental change.

### Children's Speech Patterns

Child phonology is often viewed from the adult perspective, hence the description of children's speech as fronted, harmonized, simplified, and so on. Implicit is the idea of transformed adultlike representations. As long as the transformation results in a string of phonemes readied for output, speech acquisition can be handled by an information-processing approach and construed as phonemic acquisition (see Vihman, 2017, for a review and critique of this view). When construed in this way, the learning problem is restricted to the mapping of phoneme-related speech sounds to articulatory movement. The DIVA model (Guenther, 1995; Guenther et al., 2006) instantiates this view of speech acquisition and production. The following discussion focuses on the shortcomings of this model to convey a general, developmental critique of the information-processing approach. This focus is a testament to DIVA's influence on the field and to its status as the most complete and explicit statement of an information-processing theory of speech production. Also, the original DIVA model (Guenther, 1995), though ultimately adult focused, was at least constructed to reflect the knowledge that adult behavior emerges over developmental time. This further increases the relevance of DIVA to the present discussion.

In DIVA, speech motor targets are specified as coordinates in an orosensory space. The coordinates correspond to vocal tract shapes. Speech motor goals are acoustically defined and reside in the speech sound map of the model. Linkages between the speech sound map and orosensory space are acquired during babbling. An orosensory to articulation map is established during the first phase of babbling via random articulatory movements. The speech sound map is then acquired during a second phase that relies on overt perceptual feedback to register regions in the orosensory space associated with known (i.e., perceptually acquired) language-specific sounds. Once linkages between discrete sounds and articulation have been established via orosensory space, speech production can be driven by phoneme strings that sequentially activate cells within the speech sound map.

The ease with which the DIVA model can learn to produce language-specific sequences highlights a limitation of the information-processing approach to the development of speech production: It does not take seriously the slow development of speech motor skills. Production proceeds just as in the adult once the phoneme-to-sound and sound-to-articulation mappings have been established. For example, “after babbling, the (DIVA) model can produce arbitrary phoneme strings using a set of 29 English phonemes in any combination” (Guenther, 1995, p. 598). In this way, DIVA's behavior is obviously at odds with real development. Child phonological patterns such as gliding (*leg* ➔ *weg, bread* ➔ *bwead*), stopping (*feet* ➔ *peet, house* ➔ *hout*), epenthesis (*sleep* ➔ *se-leep, green* ➔ *ge-reen*), and cluster simplification (*clean* ➔ *keen, stop* ➔ *top*) often persist until the school-age years (Stoel-Gammon & Dunn, 1985, pp. 43–46).

Although child phonological patterns can be explained within the information-processing approach by positing grammatical rules that constrain sequencing (see, e.g., Kager, Pater, & Zonneveld, 2004, and the contributions therein), the assumption that children learn via perceptual feedback to produce discrete perceptual goals in sequence incorrectly predicts that young children produce speech that is less coarticulated than adult speech (see, e.g., Guenther, 1995; Kent, 1983; Tilsen, 2014). Guenther (1995, p. 617) cites Thompson and Hixon's (1979) study on anticipatory nasal coarticulation in support of this prediction. However, the vowel midpoint measure used in that study assumes static phonemic targets that are achieved at the middle of an acoustic interval rather than the dynamic specification of movement. Flege (1988) took a different approach and measured the duration of nasalization across the entire vowel in child and adult speech. His results showed that both children and adults both open “the (velar-pharyngeal port) long before the lingual constriction for word-final /n/” (p. 533). Moreover, when vowel duration was controlled, Flege found no significant differences in the degree to which children and adults engaged in anticipatory behavior.

Guenther (1995) also cites Kent's (1983) chapter to argue that children's speech is more segmental than that of adults. This was Kent's contention, but it was not rigorously demonstrated. Instead, Kent made a qualitative comparison of F2 trajectories in 4-year-old children's and adults' production of spoken phrases. He discussed the F2 patterns in the spectrograms provided and noted that children's vowel productions appeared to be less influenced by adjacent consonantal articulations than adults' vowel productions. I found something similar in an acoustic investigation of unstressed vowels produced by 5-year-olds, 8-year-olds, and adults (Redford, 2018), but other findings were that anticipatory V-to-C effects on F1 were stronger in children's speech than in adults' speech.

In fact, findings from recent ultrasound studies on coarticulation in children's and adults' speech strongly suggest that children's speech is more coarticulated than adults' speech (Noiray, Abakarova, Rubertus, Krüger, & Tiede, 2018; Noiray, Ménard, & Iskarous, 2013; Zharkova, Hewlett, & Hardcastle, 2011, 2012; but see Barbier, 2016, for an alternative view). For instance, Zharkova et al. (2011) used ultrasound to investigate C-to-V coarticulation in school-aged children's and adults' production of /ʃV/ syllables in the frame sentence “It's a __ Pam.” They found that children's production of the palato-alveolar fricative was more influenced by the following vowel than adults' productions (see also Zharkova et al., 2011). Noiray et al. (2018) studied coarticulation degree across a wider age range and more consonantal and vocalic contrasts. Their results showed that coarticulation degree becomes weaker with age. In particular, they found that preschool children's articulation of labial, alveolar, and velar stop consonants was all more influenced by the following vowel than school-aged children's articulation of these consonants and that coarticulation degree was stronger in school-aged children's productions than in adults' productions. These and other similar results are opposite the prediction from the information-processing hypothesis that phonemes provide a basis for speech acquisition and production.

In contrast to the information-processing approach, the ecological dynamics approach to speech production predicts that children's speech is more coarticulated than adults' (Nittrouer, 1993, 1995; Nittrouer, Studdert-Kennedy, & McGowan, 1989; Nittrouer, Studdert-Kennedy, & Neely, 1996; see also Noiray et al., 2018, 2013). For example, Nittrouer (1995) hypothesized that children's early word productions are articulatory gestalts and that “the emergence of mature production skills involves two processes: differentiation and tuning of individual gestures, and improvement in coordination among gestures that compose a word” (p. 521). The hypothesis aligns well with a functional approach to child phonology, which emphasizes the communicative intent behind spoken language production and so argues for word-based analyses of children's speech sound patterns (e.g., Ferguson & Farwell, 1975; Menn, 1983; Stoel-Gammon, 1983; Vihman, 2017; Vihman & Croft, 2007; Vihman, Macken, Miller, Simmons, & Miller, 1985; Waterson, 1971). In fact, Nittrouer et al. (1989, pp. 120–121) explicitly motivated their prediction that children's speech is more coarticulated than adults' with reference to two of the articles that first introduced the idea that child phonology should take the word as its principal unit of analysis (see “setting papers” in Vihman & Keren-Portnoy, 2013). Following Ferguson and Farwell (1975), they suggested that a child's failure to appropriately generalize correct phonetic forms (e.g., [n] and [m]) from one word to another (e.g., “no” is [noʊ], but “night” is [mɑɪt], whereas “moo” is [buː]) indicated that whole words, rather than phonemes, were the targets of acquisition and also the units of production. Nittrouer et al. also referred to Ferguson and Farwell's observation of children's variable word realizations to argue for an account of word form representation as a “collection of gestures” that were inappropriately timed and so genuinely more gestalt-like than segment-like. Finally, they cited Menn's (1983) analysis of consonant harmony in her son's first words to make a point about the existence of “articulatory routines” for word production.

In summary, children's speech patterns are more compatible with the hypothesis of whole-word production than with the hypothesis of phonemic, or segmental, production. In so far as the systematic patterns of child phonology can also be explained to emerge from motoric constraints (see, e.g., Davis, MacNeilage, & Matyear, 2002; Locke, 1983; McCune & Vihman, 1987), the ecological dynamics emphasis on action-based representations is also more compatible with children's speech patterns than the information-processing emphasis on sequencing constraints derived from a child-specific grammar. For this reason, I deem holistic motoric word form representations fundamental to a developmentally sensitive theory of speech production.

### Explaining Phonological Change Over Developmental Time

As in Redford (2015), the specific proposal is that children begin to acquire holistic motoric representations, or schemas, with their attempts at first words. These schemas then provide the basic speech plan for future word productions. This proposal begs the developmental question: How do schema representations change over time as children's speech becomes more and more adultlike? Here, I argue that the information-processing assumption of separate perception and production systems is required to account for developmental change. To make this argument, let us first consider development from the ecological dynamics perspective.

In an ecological dynamics approach, learning is an attunement process (Goldstein & Fowler, 2003; Studdert-Kennedy, 1987). Unsuccessful communication destabilizes representations that encode timing relations between gestures, forcing a random walk through motor space until the word-specific timing patterns have been discovered (see, e.g., Nam et al., 2009). This mode of phonological learning implies that the temporary but systematic patterns of child phonology represent local minima in the random walk. This implication is consistent with articulatory constraint-based explanations for these patterns (e.g., Davis & MacNeilage, 2000; Davis et al., 2002; Locke, 1983; McCune & Vihman, 1987). However, similar to the constraint-based explanations, the assumption of a self-organized system based on dynamic principles predicts a universal pattern of speech development, albeit one that interacts in predictable ways with the target language. This prediction is undermined by the strong individual differences in speech development that are observed within a language (e.g., Ferguson & Farwell, 1975; Macken & Ferguson, 1981; Stoel-Gammon & Cooper, 1984; Vihman, Ferguson, & Elbert, 1986).

Ferguson and Farwell (1975) were among the first to take individual differences in development seriously and to propose, in effect, that these signal the child's control over the speech production process. The specific suggestion was that children select word forms from the adult language that they are able to produce. Word selection implies a kind of insight into the production process meted out by an executive controller—an implication that is anathema to the ecological dynamics approach. McCune and Vihman (1987, 2001) better defined the “what” of what children are able to produce when they proposed that children build up a unique set of vocal motor schemes during babbling based on individual preferences for particular patterns. Vihman (1996) then recast the notion of selection with respect to these schemas. She proposed that a schema acted as a kind of “articulatory filter” that “selectively enhances motoric recall of phonetically accessible words” (p. 142). Elsewhere, Vihman (2017) refers to resonances between the production and perception systems to explain the selective memory for phonetically accessible words. In this way, Vihman is able to explain individual differences in words and forms attempted while avoiding the homunculus problem inherent to the concept of an executive controller.

Although the idea of an articulatory filter very much implies interactions between action and perception, the specific theory of perception Vihman adopts is very clearly not a direct realist one; for example, elsewhere, Vihman is interested in the role of perceptual saliency in children's development of lexical representations (e.g., Vihman, Nakai, DePaolis, & Hallé, 2004). The notion of perceptual saliency relies on the psychoacoustic theory of speech perception that undergirds the information-processing approach of speech production, that is, a theory of perception in which the perceptual primitives are “intrinsically meaningless, simple acoustic features, such as spectral distribution patterns, bursts of band-limited aperiodic noise … into which the speech signal can be analyzed” (Best, 1995, p. 175). Why does Vihman adopt this theory? Probably because a psychoacoustic theory of speech perception provides targets of acquisition that go beyond a child's immediate abilities and so allow for directed motor learning and change (see also Menn, Schmidt, & Nicholas, 2013). More generally, a psychoacoustic theory of speech perception explains a wider variety of speech-related phenomena than a direct realist theory; for example, it accounts for categorical perception in nonhuman animals and why auditory processing constraints appear to affect the structure of phonological systems (see Diehl, Lotto, & Holt, 2004, for a review).

In summary, the observation that individual children take very different paths to acquire the same spoken language suggests a developmental process more compatible with the information-processing assumption of distinct perception and production systems than with the ecological dynamics assumption of a unified perception–action system. The developmentally sensitive theory to speech production described below further assumes that distinct production and perception systems entail a role for central perceptual feedback control in speech production.

## A Developmental Approach to Speech Production

The developmentally sensitive theory of speech production outlined in this section extends the basic idea, first outlined in Redford (2015), that adult speech production processes and representations are structured by the acquisition of spoken language. The alternative view, implicit in mainstream theory, is that adult speech production processes and representations are the targets of spoken language acquisition. As in Redford (2015), the theory assumes that the fundamental unit of production is a word. This assumption follows from the view that “the child's entry into language is mediated by meaning: and meaning cannot be conveyed by isolated features or phonemes” (Studdert-Kennedy, 1987, p. 51). Similar to an ecological dynamics approach, endogenous representations are assumed to be holistic and action based. As in Redford (2015), I call these representations *schemas,* not gestural scores or coupling graphs, to acknowledge borrowing from Vihman and McCune's theoretical work on child phonology (McCune & Vihman, 1987, 2001; Vihman & McCune, 1994) and debts to schema theory in the area of skilled action and motor control (Arbib, 1992; Cooper & Shallice, 2006; Norman & Shallice, 1986; Schmidt, 1975). These acknowledgments also signal the aforementioned embrace of certain information-processing assumptions, namely, that production and perception are distinct processes and that adults implicitly predict perceptual outcomes and use perceptual feedback to make articulatory (and whole-word) adjustments while speaking.

In addition to building on these assumptions, the developmentally sensitive theory outlined here emphasizes two distinctions: (a) the distinction between others' productions and self-productions and (b) the distinction between self-productions for oneself and self-productions for others. Self-productions provide a basis for endogenous representations. When these are for oneself, they are assumed to be exploratory and so free from association with conceptual information. In this way, they provide the basis for the nonlinguistic perceptual–motor map that is used to integrate exemplar and schema representations for production. When self-productions are for others, they are assumed to be communicative and associated with conceptual information. In this way, they provide the basis for schemas. In contrast to self-productions, others' productions provide the basis for just one type of representation—an exogenous perceptual representation associated with conceptual information. I will call this representation a *perceptual exemplar*. This label acknowledges inspiration from a class of phonetically informed phonological theories that emphasize the importance of detailed, often word-specific, acoustic–phonetic information for production (e.g., Johnson, 2007; Pierrehumbert, 2002). Perceptual exemplars provide production targets. A child cannot even attempt first words without having acquired at least a few of these from the ambient language.

The foundational assumptions enumerated above entail speech plan representations that are different from either the ecological dynamics or information-processing approaches to speech production. They also entail a different approach to phonology than the ones alluded to so far. Otherwise, the developmentally sensitive theory proposed here borrows heavily from current models of speech production and motor control. It contributes to the field by accounting for the transition from prespeech to adultlike speech in a series of steps that correspond to major developmental milestones.

### Step 1: The Perceptual–Motor Map

As in an information-processing approach to speech production, a developmental approach requires a perceptual–motor map, specifically a mapping between auditory speech and articulatory movement that is likely mediated by somatosensory information (e.g., Guenther, 1995; Guenther et al., 2006; Perkell et al., 1993). The existence of a perceptual–motor map is supported by neuropsychological findings on sensorimotor integration in different regions along the auditory dorsal stream pathway from the primary auditory cortex (= superior temporal gyrus, superior temporal sulcus) to the anterior premotor cortex (= inferior frontal gyrus; see Hickok & Poeppel, 2007). It is common to assume that the perceptual–motor map develops during the first year of life as infants engage in vocal exploration (e.g., Davis & MacNeilage, 2000; Guenther, 1995; Hickok, Buchsbaum, Humphries, & Muftuler, 2003; Kuhl, 2000; Menn et al., 2013). Following Oller (2000, pp. 165–179), I will assume that this exploration includes all prespeech vocalizations from cooing to squealing to babbling and so describes the mapping of continuous acoustic and motor dimensions, with somatosensory information at the intersection of these two. For example, it associates the frequency sweeps of squealing with continuous changes to the length and tension of the vocal folds and the amplitude-modulated frication of raspberries with the forcing of air through loosely coupled lips. It also associates static sounds, such as silence, to transient actions in the vocal tract, such as a briefly sustained oral or glottal closure. This view of the perceptual–motor map enables the gestural interpretation of acoustic form (cf. Best, 1995; see also Hickok, 2012, 2014) and so can take holistic representations as input.

Although the map develops during the prespeech period of infant vocalization, it is important to stipulate that it continues to evolve with the acquisition of speech motor skills and across the life span with the acquisition of new languages and with conformity to or disengagement from the sociolinguistic environment (see Kuhl, Ramírez, Bosseler, Lin, & Imada, 2014, for a related view). In the context of the current theory, this assumption is required to explain developmental changes that are traditionally attributed to the phonology, that is, the evolution of word forms from childlike to more adultlike. This is because the perceptual–motor map provides a source for the abstract action-based word form representations that are schemas, as described below.

### Step 2: Perceptual Word Forms and Action Schemas

Children's first words mark the onset of speech production. Word production depends on conceptual development, including the insight that adult vocalizations are referential. This insight, which occurs perhaps as early as 7 months of age (Bergelson & Swingley, 2012; Harris, Yeeles, Chasin, & Oakley, 1995), coincides with the acquisition of perceptual word forms—exemplars—from the ambient language. Bergelson and Swingley (2012) provided evidence for this claim when they used eye tracking to assess 6- to 9-month-old infants' ability to comprehend familiar nouns by discriminating between paired pictures while listening to spoken stimuli (e.g., “Can you find the *X*?” and “Where's the *X*?”). The authors reported that infants as young as 6 months of age were reliably able to discriminate a significant number of the pairs. Note that, by most accounts, perceptual attunement to the native language occurs between 6 and 10 months of age (see Vihman, 2017, for a review). Bergelson and Swingley therefore interpreted the finding to indicate that learning the sounds of a language goes hand in hand with learning its vocabulary.

**Figure 2.**
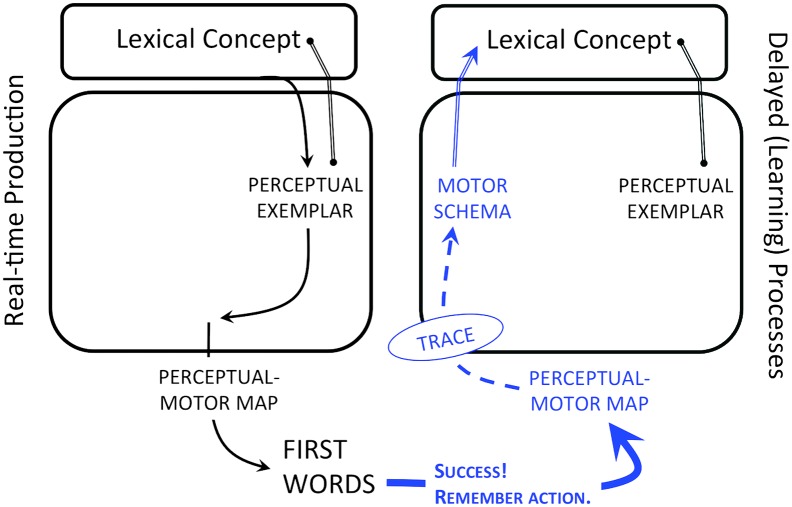
The onset of speech coincides with attempts to produce specific meanings (i.e., concepts) associated with perceptual word forms learned from the ambient language (left). Specifically, infants engage their perceptual–motor map to derive a best motoric approximation of the exogenous perceptual form or “perceptual exemplar.” The shape of the approximation will depend on how the map has been warped through vocal exploration, which itself is constrained by motor development. The motor routines used to convey specific concepts are abstracted and stored during production (right). These abstractions, or “motor schemas,” are associated with the concept attempted and so serve as one half of the phonological representation of a word. Solid lines with arrows represent feedforward processes; dotted lines with arrows represent feedback processes.

At around 12 months of age, the infant has acquired both a reasonably stable perceptual lexicon and a perceptual–motor map. The production of first words is now possible. This heralds the onset of speech production, which is imagined here as the moment when the infant, motivated to communicate a specific referential meaning, uses her perceptual–motor map to translate an exogenously derived perceptual exemplar into vocal action. As in Redford (2015), I assume that the motor routines an infant first uses to convey a particular concept are abstracted and associated with that concept when the child has succeeded in communicating the intended meaning. This abstraction is the schema. Similar to gestural scores, schemas encode routine-specific relational information between articulators across time, for example, tongue advancement during jaw opening. Similar to coupling graphs, they are the action-based word form representations. Put another way, schemas are both the phonological representation and speech plan for a given word/concept, where word is broadly construed as any conventionalized form–meaning association that is part of the child's repertoire (e.g., “uh oh” or “gimme” for “give me”). Figure 2 depicts first word production and schema abstraction.

Schemas are continually updated with production. This means that they become more abstract over time as a one-to-one relationship with a single motor routine gives way to timing generalizations that are common to all attempts of a particular word production. Note that the protracted development of articulatory timing control, which results in highly variable speech output, ensures that the schema-encoded generalizations become abstract quite quickly. Ultimately, schemas may encode little else than the number of syllables as iterations of the open–close cycle of the vocal tract and the relative durations of these cycles, plus the initial posture and direction of major articulators for each cycle. This hypothesis is consistent (or at least reconcilable) with evidence for serial timing control and frame-based plans generated in the supplementary motor area and the pre–supplementary motor area, respectively, during adult speech production (see, e.g., Bohland & Guenther, 2006; MacNeilage, 1998).

### Step 3: Onset of Perceptually Based Control

Once schemas are abstracted, they are activated with the perceptual form when a concept is selected for production. The motor and perceptual forms are integrated in the perceptual–motor map. Hickok, Houde, and Rong (2011, p. 413) adopt a similar hypothesis, albeit with an emphasis on sensorimotor integration at the level of phoneme production. They note that the hypothesis “is consistent with Wernicke's early model in which he argued that the representation of speech, e.g., a word, has two components, one sensory (what the word sounds like) and one motor (what sequence of movements will generate that sequence of sounds).” Wernicke's exact hypothesis of dual word form representations is adopted here to explain both why child forms deviate from adult forms and how the forms change over time.

With respect to children's deviant forms, schemas are assumed to initially weight production in such a way that it appears motorically constrained. The weighting is the result of a very small productive vocabulary, which serves to entrench particular trajectories through motor space. For a while, this entrenchment may even limit the child's ability to form new motor trajectories. At this stage, children's productions of novel words may appear more template-like than in first word production. In Vihman and Croft's (2007, p. 696) words, “the child (implicitly) impos(es) one or more preexisting templates, or familiar phonological patterns, on an adult form that is…similar to those patterns.”

Around 18 months of age, significant vocabulary expansion results in a developmental shift away from forms that suggest production constraints and toward those that suggest perceptual ones due to increasing homophony among expressive word forms (Redford & Miikkulainen, 2007). This shift heralds the next critical step in the evolution of speech production: a newfound focus on how self-productions should sound. The onset of predictive encoding (state feedback control) emerges from this focus.

**Figure 3.**
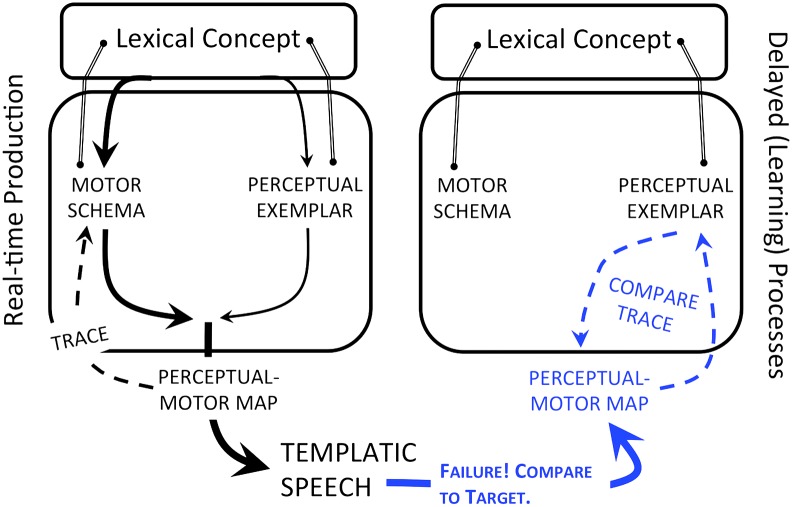
Following early word production, the next major developmental change is hypothesized to occur when motorically driven homophony begins to threaten the young child's ability to effectively communicate. At this stage, the child begins to focus on how words should sound. As a result, production shifts from an entirely feedforward process to one where feedforward routines are adjusted to match perceptual representations. The adjustment process, carried out through interactions between the endogenous perceptual–motor map and the repository of exogenous word form representations or “perceptual exemplars,” sets the stage for state feedback control, which nonetheless begins with a delayed comparison between the perceptual trace and target—absent adjustment (left). Solid lines with arrows represent feedforward processes; dotted lines with arrows represent feedback processes.

In particular, the proposed process by which the 18-month-old infant begins to forge new paths through motor space takes as its inspiration the hierarchical state feedback control model of production (Hickok, 2012, 2014; Hickok et al., 2011), where state feedback control is described as having two functions. The first is to adjust motor commands so that the articulators reach desired perceptual targets; the second is to use external feedback to update the representations that guide speech. In the present proposal, both functions are thought to emerge with a communication-driven shift in production toward better matching of endogenously derived motor forms to exogenously derived perceptual forms. Furthermore, Function 2 is proposed to drive Function 1 in that Function 1 may begin as a delayed comparison between the perceptual trace of a production and the intended target, absent any motor adjustments (see Figure 3).

How might a delayed matching process evolve into real-time state feedback control? One possibility is that the matching process creates a bidirectional connection between the exogenously derived exemplar targets and the perceptual–motor map, where the connections between motor routines and perceptual patterns are already robust and bidirectional. Now, the perceptual outcomes of schema-associated routines can be matched in real time against perceptual exemplars. Any discrepancies between the expected self-outcomes and other-based representations could force new paths through motor space by stretching entrenched motor routines in the direction of the exogenously derived perceptual form.

### Step 4: Self-Monitoring

Speech production does not become adultlike until children begin to externally monitor their own speech and consciously recognize its divergence from (chosen) adult norms. The evidence suggests that this may not occur until around the age of 4 years. In particular, feedback perturbation experiments with young children suggest that perceptual input plays little role in speech production before the age of 4 years; for example, toddlers neither immediately compensate nor adapt over time with articulatory changes to their vowel productions when hearing spectrally perturbed alterations of their own speech during a word production task (MacDonald, Johnson, Forsythe, Plante, & Munhall, 2012). At the age of 4 years, children begin to compensate but do not adapt over the long term to perturbed feedback (MacDonald et al., 2012; Ménard, Perrier, Aubin, Savariaux, & Thibeault, 2008); for example, Ménard et al. showed that 4-year-old children return immediately to preferred productions after compensating online to an articulatory perturbation. Failures to adapt suggest that, although 4-year-old children may use auditory information to help guide speech production, they do not yet use external feedback to update existing production representations and processes. Still, the ability to adapt appears to emerge soon after 4 years of age in typically developing children (Terband, Van Brenk, & van Doornik-van der Zee, 2014).

Psycholinguistic evidence is consistent with the hypothesis that self-monitoring emerges late in the preschool years during spoken language development. For example, preschool children understand unfamiliar adult speech better than their own unadultlike speech (Dodd, 1975). In addition, self-initiated speech repairs increase over developmental time, with many fewer repairs observed in the speech of 5-year-old children than in the speech of older school-aged children (Evans, 1985; Rogers, 1978). Moreover, if we imagine the self-monitoring process as one where the speaker must identify particular discrepancies between what they intended to produce and what they actually produced, then its slow development is consistent with the slow development of selective attention (see, e.g., Plude, Enns, & Brodeur, 1994; Wellman, Cross, & Watson, 2001). The speculation here is that selective attention to one's own speech is motivated also by a developing self-concept. When the child begins to appreciate those aspects of his or her own speech that signal an undesired social distance between himself or herself and others, he or she shifts his or her attentional focus to identifying discrepancy between how he or she sounds and who he or she wants to sound like. This motivates a final marked disruption of entrenched motor routines in service of better approximating the exogenously derived exemplars.

**Figure 4.**
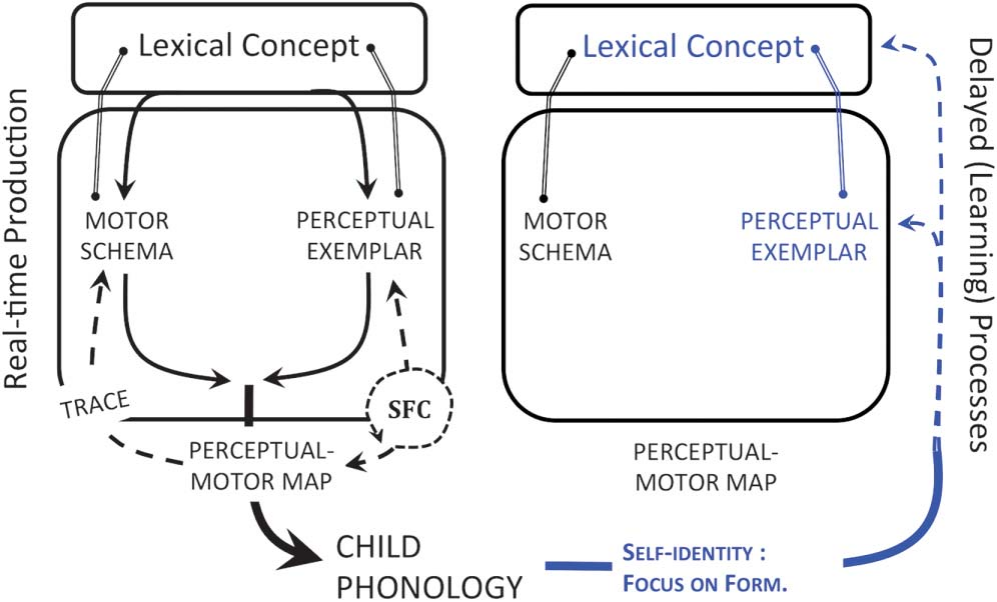
During the preschool years, children begin to self-monitor based on external perceptual feedback to identify deviations between how they sound and who they want to sound like. The perceived deviations highlight aspects of the stored perceptual representations, driving the perceptual–motor mapping and resulting endogenous motoric representations (i.e., schemas) ever more toward matching exogenous perceptual goals (i.e., exemplars). Solid lines with arrows represent feedforward processes; dotted lines with arrows represent feedback processes.

Self-concept emerges with theory of mind during the preschool years (see Symons, 2004). Self-identity, which is part of the self-concept (Baumeister, 1999; Gecas, 1982), manifests in speech with socio-indexical marking. For example, VOT for stops varies differently as a function of gender across languages (Li, 2013; Oh, 2011; Whiteside & Irving, 1998), suggesting social as opposed to physiological reasons for this speech production difference. How does the child acquire female- versus male-gendered speech? The suggestion here is that a burgeoning sense of identity leads the child to selectively attend to those adult productions he or she is most interested in approximating. In identifying a discrepancy between how they sound and who they want to sound like, children may highlight exemplars associated with those individuals, thereby highlighting aspects of the perceptual form that need special attention in production. At the same time, self-monitoring focuses more attention on the perceptual consequences of one's own speech, which further increases the weight of exemplars in the production process, thus pushing motor routines and resulting schema ever more in the adult direction (see Figure 4).

Thus, the full proposal is that, during the preschool years, socially directed listening induces changes in speech production through a self-monitoring–led shift toward perceptually weighted production. Prior to this point, self-productions are (unconsciously) heard as being the same as other productions. Consider, for example, the toddler who points to a picture of a fish in a picture book and utters “fifth,” to which the parent responds “fifth?” and the child answers, “No, fifth!” (see Menn, 1983). Updates to both the perceptual–motor map and schema representations follow from this shift, soon resulting in adultlike representations. This proposed final stage in the development of speech production is consistent with the evidence that socio-indexical information, such as gender-specific use of phonetic features, begin to emerge in children's speech around the age of 4 years (see Foulkes & Docherty, 2006, pp. 422–424). This observation brings us back to an earlier one that closes the gap between work in speech motor control and real-world speaker behavior, that is, the observation that participants' behavior in auditory feedback perturbation experiments resembles phonetic convergence, normally understood as a socially driven behavior meant to lubricate interactions between interlocutors.

## Discussion

Current approaches to speech production aim to explain adult behavior and, in so doing, frequently make at least some assumptions that, when taken to their logical conclusion, fail to adequately account for how the system develops. This failure is problematic from a developmental perspective. According to this perspective, the representations and processes of adult speech and language should emerge from the developmental process (for a similar view, see Menn et al., 2013; Vihman & Croft, 2007).

Development is particularly relevant for theories of speech production because of the paradox of early speech onset despite slowly developing speech motor control. Here, this paradox was taken to suggest the working hypothesis that feedforward processes mature earlier than central feedback control processes in speech production. This hypothesis structured a developmentally sensitive theory of speech production that was elaborated in stages, with each stage building on the previous one. The stages proposed were designed to accommodate developmental patterns. At the same time, developmental patterns were given new meanings and grouped in novel ways by the working hypothesis. The accommodation of speech production theory to developmental findings and vice versa results in many new testable hypotheses that could motivate future empirical work and usher in new knowledge and even new clinical practice. For example, the hypothesis that perceptual–motor integration relies on the development of a nonlinguistic perceptual–motor map suggests that therapeutic uses of speech sound practice should cover as broad a range of sound combinations as possible. By hypothesis, these sound combinations need not be tied to lexical content and so the therapy could involve a fun and silly random sound sequence–generating game using, say, magnetic letters that could be arranged and then rearranged on a board. Such a game would allow the set of possible sound combinations in a language to be more fully explored than is possible when that set is constrained by picturable words in the language. The benefits of this therapy for generalization to novel or known word production could be tested against current therapies where speech sound practice typically involves the use of visual props to elicit specific lexical items. Intriguingly, this idea echoes, to some extent, Gierut's (2007) differently motivated contention that words with complex speech sound sequences allow for better generalization of treatment in children with phonological disorder than words that have simple phonological structure.

The hypothesized disassociation of the perceptual–motor map and perceptual exemplar representation of word forms also has implications for the clinical assessment of speech sound disorder. For example, when this hypothesis is taken together with the idea that articulatory change is motivated by weighting perceptual exemplar representations more heavily during production, it suggests that the aforementioned fun and silly random sound sequence–generating game could be used to supplement a comprehensive evaluation of speech sound disorder. Performance in the game could help diagnose whether the articulation problem is due to a poorly developed perceptual–motor map or to poorly specified perceptual exemplars. The diagnosis would then lead to therapy that focuses either on speech sound practice or on developing perceptual exemplars. Finally, the theory-dependent hypothesis that perceptual weighting of production is driven in part by the emergence of a self-concept and the ensuing selective attention to self-productions suggests not only a testable hypothesis regarding the development of convergence behaviors in spoken language interactions but also a novel way to understand the absence of convergence behaviors and mild segmental speech sound disorders in individuals on the autism spectrum.

**Figure 5.**
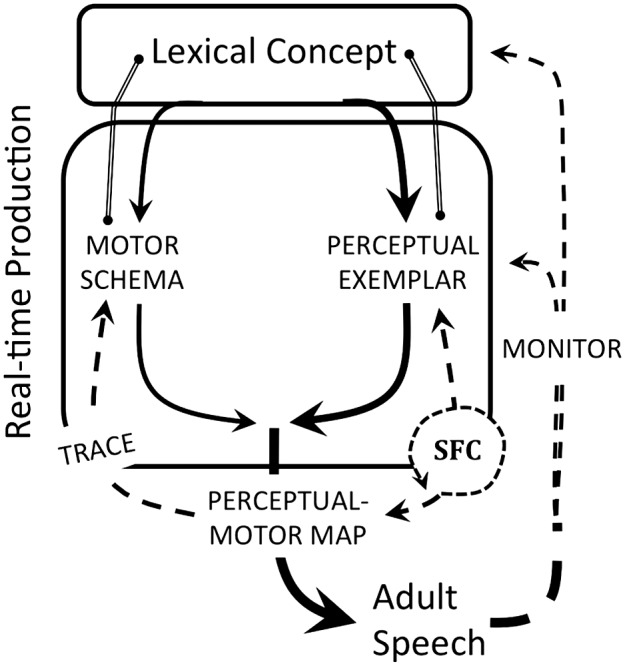
The adult model of speech production implied by the developmental model outlined in this review article. Solid lines with arrows represent feedforward processes; dotted lines with arrows represent feedback processes. The linkages between the repository of lexical concepts and motor schemas and between lexical concepts and perceptual exemplars represent the conceptual and phonological aspects of the lexicon.

Another major implication of the developmentally sensitive theory elaborated in this review article is a new adult model of speech production. This model, illustrated in Figure 5, incorporates insights from many existing theories. Some of these insights were explicitly acknowledged in the preceding text; others were merely implied. For example, the reference to “self-monitoring” indicates an acceptance of the evidence in favor of this well-established hypothesis (see Postma, 2000, for a review). Otherwise, the model diverges from most adult-focused theories in assuming distinct action- and perception-based representations (though see Hickok, 2012, 2014). This aspect of the model provides a framework for understanding phenomena that have been traditionally ignored in adult-focused theories of speech production. For example, the model very obviously allows for the different possible speaking modes that are thought to correspond with speaking style differences specifically, one mode wherein the motor pathway is emphasized over the perceptual pathway—this is Lindblom's (1990) hypo or system-oriented mode, one mode wherein the reverse occurs—this is Lindblom's hyper or output-oriented mode (shown); and a mode mode wherein the two pathways are in equilibrium—this is likely the default mode.

The implied adult model shown in Figure 5 also diverges from information-processing theories in assuming that holistic phonological representations serve as speech plan representations. This developmentally sensitive aspect of the model is not immediately compatible with the evidence for sublexical units in productions, including the speech error data that have long been used to argue for the psychological reality of a phonological encoding process. The developmentally sensitive adult model automatically fails if it cannot account for these data. Accordingly, we are currently pursuing the hypothesis that discreteness emerges at the level of the perceptual–motor map (Davis & Redford, 2019). More specifically, we have formally defined the perceptual–motor map as a linked set of experienced perceptual and motor trajectories that are time-based excursions through speaker-defined perceptual and motor spaces. By hypothesis, nodes appear where motor trajectories intersect in motor space, creating perceptually linked node-delimited paths that can be recombined. Though weighted in the direction of already experienced paths, exemplar-driven novel word production picks new trajectories through motor space by deforming existing node-delimited paths in systematic ways. These new trajectories may intersect existing trajectories or go on to be intersected themselves. In this way, motor space is reticulated with vocabulary acquisition, and discrete speech motor goals emerge absent discrete phonological representations. In future work, we will investigate how this view of discreteness might account for the speech error data. Our initial hypothesis is that these arise from the competing motoric and perceptual pressures of schema and exemplar integration during speech production.

## Conclusion

Theories of spoken language production provide frameworks for understanding developmental speech sound disorders. Even the distinction between motor speech, articulation, and phonological disorders reflects this fact. In so far as the types of interventions chosen to address a disorder follow from how the disorder is understood, theory informs practice. This is as it should be. However, the relationship between theory and practice should also motivate a reconsideration of theory when it fails to address a problem that is relevant to practice. The problem of development clearly falls into this category. A major aim of this review article was to show that current adult-focused approaches to speech production fail to address the paradox of slow developing speech motor control despite early speech onset because they depart from perspectives that are not developmental. A developmental perspective assumes change over time, and those who adopt it focus on explaining how this change occurs. A second major aim of this review article was to show how a commitment to this perspective leads to a theory of speech production that is different in many respects from existing theories. Thus, even if the various ideas presented herein are dismissed after testing, the conclusion should be that a developmental approach to understanding speech production should be pursued if theory is to be useful for practice.
